# *Eugenia uniflora* L.: Analysis of Chemical Profile and Cytotoxic Action on Tumor (HeLa) and Non-Tumor Cells (NIH/3T3)

**DOI:** 10.3390/ph18081199

**Published:** 2025-08-14

**Authors:** Letícia M. R. Pescinelli, Milena França Longue, Giovana G. F. V. de Oliveira, Júlio C. Thurler-Júnior, Thiago S. Charret, Thalya S. R. Nogueira, Mariana T. M. Pereira, Ivo J. C. Vieira, Lucas S. Abreu, Vinicius D. B. Pascoal, Aislan C. R. F. Pascoal

**Affiliations:** 1 Research Laboratory of Natural Products and Bioactive Molecules, (Lab Nat—UFF) Nova Friburgo Health Institute, Fluminense Federal University—UFF, Nova Friburgo 28625-650, RJ, Brazil; leticiapescinelli@id.uff.br (L.M.R.P.); milenalongue@id.uff.br (M.F.L.); gi_fortes@id.uff.br (G.G.F.V.d.O.); thiagosardou.nf@hotmail.com (T.S.C.); marianatmartinsp@gmail.com (M.T.M.P.); viniciuspascoal@id.uff.br (V.D.B.P.); 2 Multi-User Biomedical Research Laboratory, Nova Friburgo Health Institute, Fluminense Federal University—UFF, Nova Friburgo 28625-650, RJ, Brazil; juliothurler@id.uff.br; 3 Laboratory of Chemical Sciences, Center for Exact Sciences and Technology, State University of North Fluminense Darcy Ribeiro—UENF, Campos dos Goytacazes 28013-602, RJ, Brazil; thalyasrnogueira@gmail.com (T.S.R.N.); curcino@uenf.br (I.J.C.V.); 4 Natural Products Chemistry Laboratory, Institute of Chemistry, Fluminense Federal University—UFF, Niteroi 24210-201, RJ, Brazil; abreu_lucas@id.uff.br

**Keywords:** natural products, cancer, myrtaceae, biological activities

## Abstract

**Objectives:** This study analyzed the antiproliferative potential of *Eugenia uniflora* L. leaf extracts against cervical cancer and non-cancerous cell lines. **Methods:** The extracts were prepared by maceration using hexane (EUH), dichloromethane (EUD), and ethyl acetate (EUA). Their cytotoxic potential was evaluated through MTT assays, wound healing assays, and flow cytometry. To identify classes of secondary metabolites, total phenolic and flavonoid contents were quantified using spectrophotometric methods, and individual metabolites were tentatively identified by LC-MS/MS. **Results:** EUH, EUD. and EUA exhibited cytotoxicity in HeLa cells, with IC_50_ values of 63.03 μg/mL, 33.79 μg/mL, and 38.38 μg/mL, respectively. Due to their lower IC_50_ values, the EUD and EUA fractions were selected for further investigation. EUA and EUD inhibited cell migration at all the time points tested and altered the cell cycle. Twenty-eight compounds were tentatively identified in *E. uniflora* L. leaf extracts based on the interpretation of their fragmentation patterns and molecular formulas obtained from mass spectrometry. **Conclusions:** The EUD and EUA extracts appear to modulate the metabolism of cervical cancer cells, leading to cell cycle arrest and inhibition of cell migration. Flavonoids and other phenolic compounds are likely responsible for these observed biological effects.

## 1. Introduction

In modern times, natural products remain essential to advancements in healthcare, particularly in the pharmaceutical field. They serve as sources for new drugs, either through crude extracts or by enabling the synthesis of medicines from isolated active compounds [1]. Furthermore, according to a review by Newman and Cragg, over the past 40 years, natural products have become increasingly significant in drug development. Between 1981 and the end of 2019, approximately 49.5% of the approved pharmacological drugs were natural products, derived from them, or contained pharmacophores related to them [2].

Within this context, natural products, particularly those derived from plants, have been widely explored in the search for new treatments, such as chemotherapeutic agents. Approximately 50% of antitumor drugs are derived from natural products [1,3]. Phytochemical compounds from medicinal plants, including polyphenols and flavonoids, are considered promising agents in cancer treatment, as they can modulate the expression of oncogenes and exhibit antiproliferative, antineoplastic, pro-apoptotic, and anti-inflammatory activities [4].

Cervical cancer is caused by persistent infection with the *Human Papillomavirus* (HPV). HPV can infect the skin and mucous membranes. Of the approximately 200 types of HPV, at least 12 are considered oncogenic, particularly types 16 and 18. These two are associated with a higher risk of persistent infection and the development of precursor lesions, which, if not correctly diagnosed and treated, can progress to cancer. Types 16 and 18 are responsible for about 70% of cervical cancer cases [5]. Cervical cancer is the fourth most common cancer among women worldwide, with over 660,000 new cases and more than 300,000 deaths reported in 2022, making it the fourth leading cause of cancer-related death in women [6].

Although conventional chemotherapeutic agents remain a cornerstone in cancer treatment, their efficacy is often limited by several critical drawbacks. A primary concern is their lack of selectivity for malignant cells, which leads to collateral damage in healthy, rapidly dividing cells, such as those in the gastrointestinal tract, bone marrow, and hair follicles. This non-specificity results in adverse effects, including nausea, anemia, immunosuppression, mucositis, and alopecia, significantly impairing patients’ quality of life and treatment adherence [7,8]. Furthermore, the prolonged use of chemotherapy can trigger the development of multidrug resistance (MDR) in tumor cells. This phenomenon is often mediated by overexpression of efflux transporters such as P-glycoprotein (P-gp), alterations in drug targets, enhanced DNA repair mechanisms, and evasion of apoptosis pathways, ultimately leading to therapeutic failure [9,10].

In addition, tumor heterogeneity and the dynamic nature of cancer progression complicate treatment responses, as subpopulations of resistant cells can survive and repopulate the tumor mass. Combined with the emergence of aggressive phenotypes and the adaptation of the tumor microenvironment, these factors make long-term disease control challenging [11]. These limitations highlight the urgent need to discover and develop novel anticancer agents that are more selective for tumor cells, possess lower toxicity profiles, and are capable of overcoming or bypassing resistance mechanisms. In this context, bioactive compounds derived from medicinal plants have garnered increasing attention, as they often exhibit multiple mechanisms of action, lower systemic toxicity, and potential synergistic effects when used alongside conventional therapies [12,13].

*Eugenia uniflora* L., a member of the Myrtaceae family, is commonly known as “Pitangueira,” with its fruit referred to as “Pitanga” or “Red Cherry.” Native to Brazil, it is cultivated in various regions around the world [14]. Widely used in traditional medicine, tea made from its leaves is often used to treat inflammation, digestive issues, fever, rheumatism, and hypertension [15].

Several studies have reported that the leaves of *E. uniflora* possess a range of therapeutic effects, including antibacterial, antifungal, antiviral, anti-inflammatory, hypotensive, antioxidant, antitumor, diuretic, and hypoglycemic activities [14,15,16]. These therapeutic actions are likely related to the presence of various secondary metabolites such as terpenes, flavonoids, tannins, leucoanthocyanidins, steroids, and triterpenes [16]. In the present study, *E. uniflora* was investigated due to its recognized pharmacological potential and the limited mechanistic data available regarding its antitumor properties. Notably, this is the first study to evaluate the effects of *E. uniflora* extracts on cervical cancer cells through cell migration assays and cell cycle analysis by flow cytometry, providing deeper insights into its potential antiproliferative mechanisms.

Given the global impact of cervical cancer and the limitations of current chemotherapeutic drugs, the search for new, effective anticancer compounds is of the utmost importance. Medicinal plants such as *E. uniflora* represent a promising source. Therefore, this study aimed to evaluate the antiproliferative and antitumor potential of *E. uniflora* leaf extracts on cervical cancer cells (HeLa).

## 2. Results

### 2.1. Chemical Profile

To quantify the total soluble phenolic content in *E. uniflora* extracts, the Folin–Ciocalteu colorimetric method was employed, a widely accepted reference technique for evaluating phenolic compounds in natural products [17]. In this assay, polyphenols act as reducing agents, reacting with the Folin–Ciocalteu reagent to produce a blue coloration, the intensity of which correlates with the phenolic content [18].

Flavonoid content was determined using a spectrophotometric method based on the formation of stable complexes between the carbonyl groups of flavonoids and Al^3+^ ions in a methanol-based solvent system [19]. The results for the total soluble phenolic and flavonoid contents are summarized in Table 1. Phenolic compounds were more abundant in the ethyl acetate extract (EUA), which contained 45.94 μg gallic acid equivalents (GAE)/mg, compared to the dichloromethane extract (EUD), which contained 10.71 μg GAE/mg. Conversely, the flavonoid content was higher in the dichloro-methane extract, with 23.82 μg quercetin equivalents (QE)/mg, whereas the EUA extract contained 14.22 μg QE/mg.

**Figure 1:**
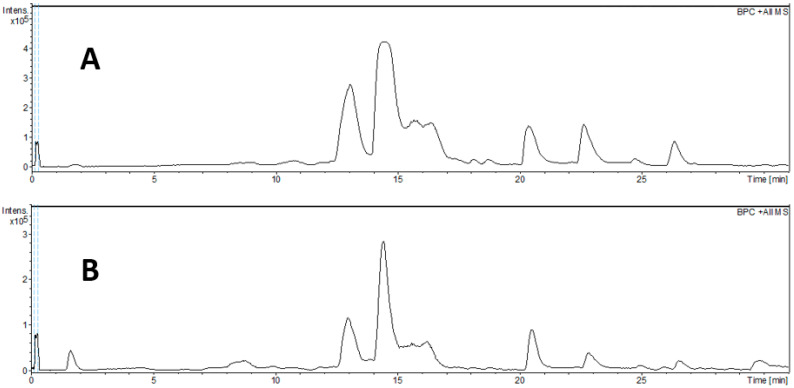
BPC chromatogram of dichloromethane (**A**) and ethyl acetate (**B**) extract from leaves of *Eugenia uniflora* by HPLC-DAD-qTOF.

A total of 28 compounds were tentatively identified in the *E. uniflora* leaf extracts through analysis of their fragmentation patterns and molecular formulas obtained from high-resolution mass spectrometry (HRMS/MS) (Figure 1). Published data were also used to support compound annotation. Retention times, mass spectral data, and peak assignments obtained from both positive and negative ionization modes are summarized in Table 2.

Regarding the annotation of phenolic compounds (**1**–**5**), precursor ions with *m*/*z* 191.0560, 169.0144, and 300.9981 were attributed to quinic acid, gallic acid, and ellagic acid, respectively. Precursor ions with *m*/*z* 331.0671 and 361.0768 corresponded to galloyl hexoside and galloylquinic acid. Additionally, the ion at *m*/*z* 463.0877, showing a characteristic loss of a glycosidic residue (146 Da), was attributed to the flavonoid myricetin-O-rhamnoside (compounds **1**–**2**). These phenolic compounds have been previously reported in *E. uniflora* [20,21]. Compounds **6**–**28** were tentatively associated with rearranged and rare sesquiterpenoids. Chen and collaborators previously reported the isolation and identification of eugenunilones A–H and eugenilones A–N using NMR, HRMS, and other spectroscopic techniques [22,23]. Based on the molecular formulas observed for compounds **6**–**28**, the presence of sesquiterpenes, including mono- and diacetylated derivatives, was proposed. Although MS/MS data for these compounds were not available in the literature for comparison, we included our own MS/MS data in Table 2 to support tentative identification. Notably, compounds **8**–**10**, **18**–**19**, and **25**–**28** were not reported among the sesquiterpenes isolated by Chen et al. and thus remain unassigned [22,23]. These compounds may represent novel sesquiterpenes, warranting further studies for isolation and structural elucidation.

### 2.2. Cell Viability

**Figure 2:**
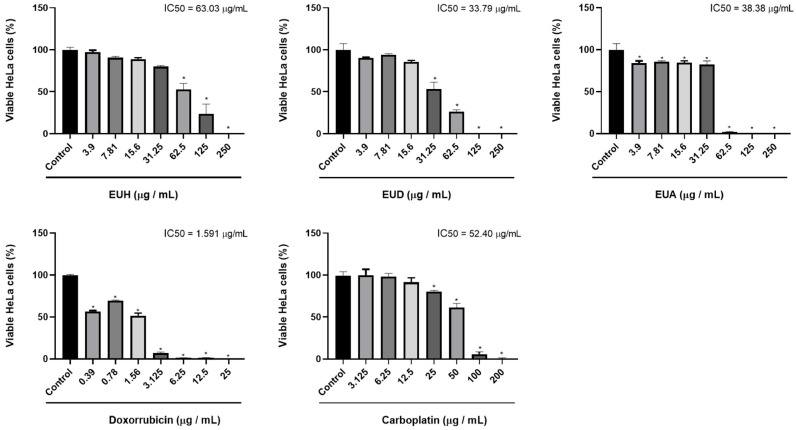
Results of the HeLa cell viability assay using the MTT method, in percentage (%), through the bar graph, comparing cells of the HeLa cell line after receiving the respective treatments: EUH, EUD, EUA, doxorubicin, and carboplatin. One-way ANOVA was used for statistical analyses, followed by Tukey’s test, considering the control group (vehicle) with 100% cell viability. “*” indicates the sample concentration at which there was a statistically significant difference (*p* < 0.05) when compared to the control (vehicle).

The viability of HeLa (cervical cancer) and NIH/3T3 (murine fibroblast) cells following treatment with *E. uniflora* leaf extracts was assessed using the MTT colorimetric assay, which estimates cellular metabolic activity. This assay determined the concentration required to inhibit 50% of cell viability (IC_50_). Both cell lines were treated with a negative control (vehicle); positive controls (doxorubicin and carboplatin); and the *E. uniflora* extracts EUH (hexane), EUD (dichloromethane), and EUA (ethyl acetate) (Figure 2).

A statistically significant reduction in cell viability was observed in the HeLa cells compared to the negative control (considered 100% viability). The EUH extract showed a significant difference only at concentrations above 62 μg/mL, with an IC_50_ of 63.03 μg/mL, indicating moderate cytotoxicity. The EUD extract significantly reduced viability at concentrations between 31.25 and 250 μg/mL, with an IC_50_ of 33.79 μg/mL. The EUA extract exhibited cytotoxic effects at all the tested concentrations, with a statistically significant difference from the negative control and an IC_50_ of 38.38 μg/mL. Based on these results, the EUD and EUA extracts were selected for further experiments due to their IC_50_ values being close to 30 μg/mL. Regarding the positive controls, both doxorubicin and carboplatin reduced HeLa cell viability at all the tested concentrations, with IC_50_ values of 1.591 μg/mL and 52.40 μg/mL, respectively, and the chemotherapeutic carboplatin shows statistically significant results only above the concentration of 25 μg/mL.

**Figure 3:**
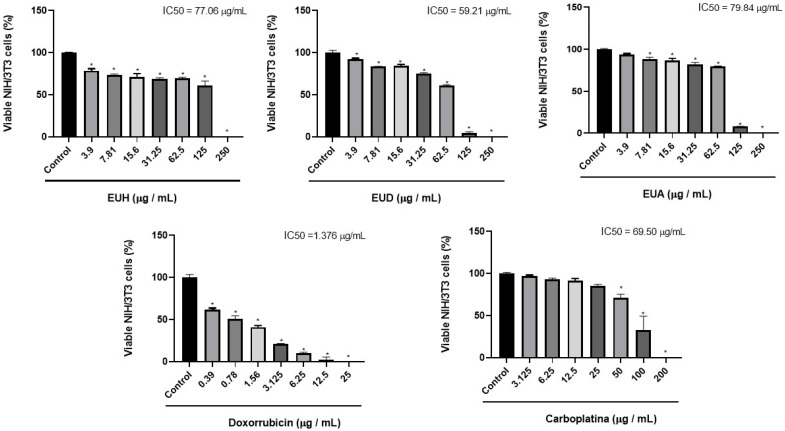
Results of the cell viability assay in the NIH/3T3 murine cells using the MTT method, in percentage (%), through the bar graph, comparing cells of the HeLa cell line after receiving the respective treatments: EUH, EUD, EUA, doxorubicin, and carboplatin. One-way ANOVA was used for statistical analyses, followed by Tukey’s test, considering the control group (vehicle) with 100% cell viability. “*” indicates the sample concentration at which there was a statistically significant difference (*p* < 0.05) when compared to the negative control.

In the NIH/3T3 non-tumor cell line, the extracts of *E. uniflora* L. showed cytotoxic effects only at concentrations above 100 µg/mL. At lower concentrations (3.9 to 62.5 µg/mL), cell viability remained above 70%, with no significant reduction observed. Thus, it was not possible to accurately calculate the IC_50_ value, as the dose–response curve did not reach 50% inhibition at the tested concentrations. The logistic model typically used to estimate IC_50_ requires points distributed around the 50% viability range for a statistically reliable fit. Therefore, the IC_50_ values obtained were calculated by extrapolation, and these data suggest that the extracts have low toxicity in non-tumor cells, especially at doses below 100 µg/mL. Doxorubicin also inhibited metabolic activity in non-tumor cells at all concentrations, with an IC_50_ of 2.321 μg/mL. In contrast, carboplatin exhibited cytotoxic effects only at the three highest concentrations tested, resulting in an IC_50_ of 38.32 μg/mL (Figure 3).

### 2.3. Selectivity Index (SI)

The SI values were calculated from the IC_50_ values obtained through the colorimetric assay of metabolic cellular activity, MTT, in tumor and non-tumor cells (Table 3).

### 2.4. Cell Migration Assay—Scratch Assay

**Figure 4:**
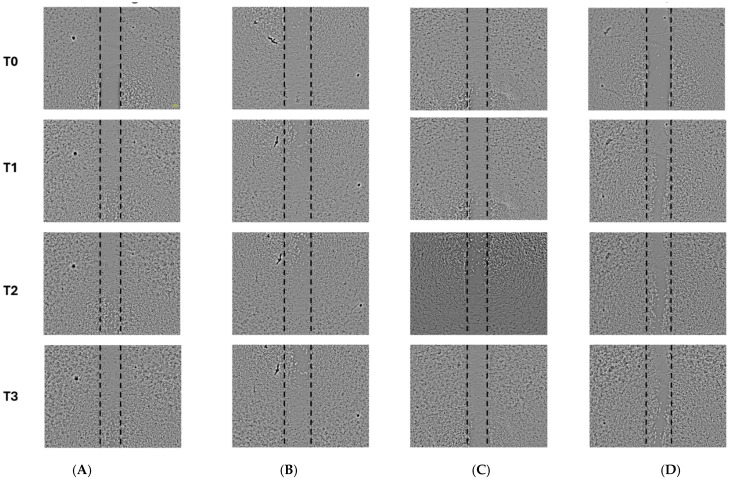
Microscopic images were obtained using an EVOS microscope (10× objective) during the assay: (**A**) cells that did not receive any treatment (negative control); (**B**) cells treated with EUD; (**C**) cells treated with EUA; (**D**) cells treated with doxorubicin. Photographs were captured before treatment (T0) and after 12, 18, and 24 h of treatment (T2, T3, and T4, respectively). The doses used were EUD 7.5 μg/mL, EUA 10 μg/mL, and 0.375 μg/mL doxorubicin.

As the hexane fraction presented an IC_50_ above 60 μg/mL, demonstrating moderate activity, the studies focused on the ethyl acetate and dichloromethane extracts. By analyzing the scratch area in the confluent monolayer, the percentage of free area (%) in each well was quantified, allowing the evaluation of in vitro cell migration following treatment with the vehicle (negative control), EUD and EUA extracts, and doxorubicin (positive control). Based on the images captured at the designated time points, it was evident that the EUD and EUA extracts, at the tested concentrations, effectively inhibited cell migration compared to the negative control, with EUA showing the most pronounced effect. Notably, the positive control (doxorubicin), at the applied concentration, did not significantly inhibit tumor cell migration (Figure 4).

**Figure 5:**
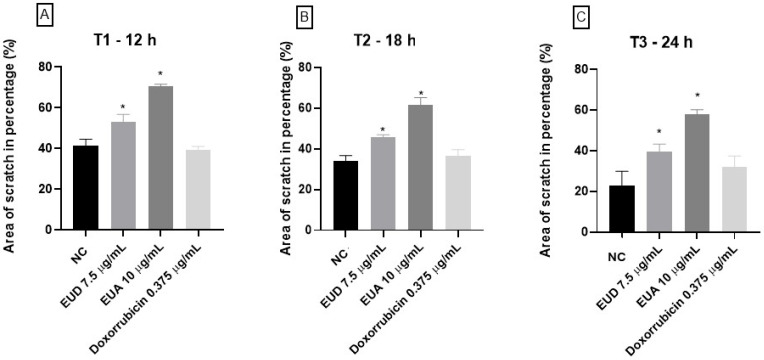
Statistical analysis of the percentage (%) of the area free of cell migration, in a bar graph, at T1, T2, and T3, representing 12 (**A**), 18 (**B**), and 24 (**C**) hours, respectively, after the application of the treatment, in which “*” represents a statistically significant difference when compared to the negative control (NC). One-way analysis of variance (ANOVA) was used for the analyses, followed by the multiple comparison test, considering *p* < 0.05.

At all three recorded time points (T1: 12 h, T2: 18 h, and T3: 24 h), both the EUD and EUA extracts demonstrated statistically significant differences compared to the negative control. The EUD treatment resulted in 53.24% free area at T1, 45.80% at T2, and 39.69% at T3. In contrast, the EUA extract showed greater inhibition of cell migration, with 70.73% free area at T1, followed by 61.41% and 58.09% at T2 and T3, respectively. The doxorubicin treatment did not significantly differ from the negative control, with free areas of 39.51%, 36.49%, and 32.17% at the respective time points (Figure 5).

### 2.5. Cell Cycle Arrest Analysis Using Flow Cytometry

The cell cycle is the set of phases a cell goes through to duplicate itself, giving rise to two new cells. In eukaryotic cells, the cell cycle is divided into three main phases: interphase, Mitotic Phase (M Phase), and cytokinesis. These phases are of paramount importance for the functioning of the cell; errors in these processes can lead to cell death or even the development of tumor cells [24].

The interphase is divided into Phase G1 (in G1 cells are diploid, their DNA content is referred to as 2n), where the cell prepares for the duplication of its genetic material, and the cell grows and performs its normal functions, accumulating energy and nutrients for the next cycle of cell division. The S phase of the cell cycle is the period of DNA synthesis, in which the cell’s DNA is duplicated. Replication increases the DNA content of the cell from 2n to 4n, so the cells in S have DNA contents ranging from 2n to 4n. It is the stage of preparing the cell for division as it grows and duplicates its DNA. During G2, the cell synthesizes RNA and proteins, preparing for division. DNA content remains at 4n for the cells in G2 and M, decreasing to 2n after cytokinesis. In addition, the synthesis of molecules essential for division, such as centrioles, occurs [25]. With the alterations in the DNA content and its fragmentation, compared with a negative control, it is possible to observe in which phase of the cell cycle the compounds being tested can act.

Intracellular signals act by modifying the activity of the primary regulators of the cell cycle. There is a certification that the previous phase has been completed correctly, so that one can move on to the next phase. Cyclins are a group of essential proteins that regulate the cell cycle, and there are four basic types found in humans and most other eukaryotes: G cyclins, G cyclins, S, S cyclins, and M cyclins. Each cyclin is associated with a particular phase, transition, or set of phases in the cell cycle and helps drive the events of that phase [26,27].

To cause the cell cycle to move forward, a cyclin must activate or deactivate many target proteins within the cell. These events can occur when cyclin associates with a family of enzymes called cyclin-dependent kinases (Cdks). A Cdk alone becomes inactive, but binding with a cyclin activates it, making it a functional enzyme and allowing it to modify target proteins within the cell [28].

Cancer is a disease caused by uncontrolled cell division, and its development and progression are normally linked to a series of changes in the activity of cell cycle regulators. Thus, when a compound can stop the cell cycle of cancer cells, this can be a positive point in inhibiting the progression and development of the tumor. Some chemotherapy drugs act at specific points in the cell cycle, and in our results, we can observe that EUD and EUA can behave differently in the cell cycle of HeLa cells.

**Figure 6:**
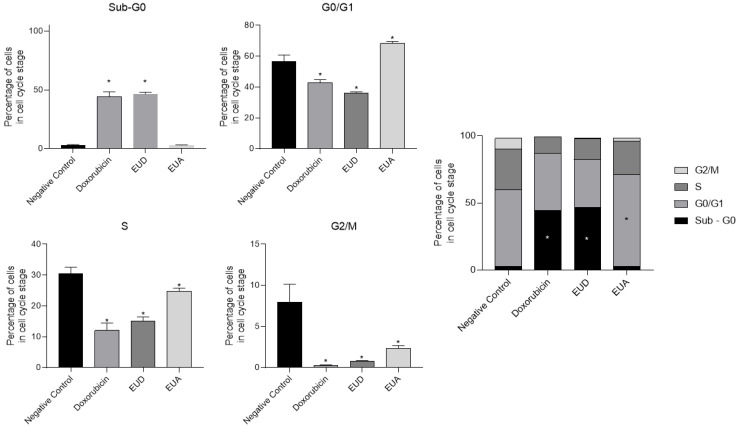
Cell cycle distribution of HeLa cells after treatment. HeLa cells (HeLa) were treated with vehicle (negative control), doxorubicin (positive control), EUD, and EUA: the percentage of cells in the G0/G1 phase is presented as the mean ± SD; the percentage of cells in the G2/M phase is presented as the mean ± SD; the percentage of cells in the S phase is presented as the mean ± SD; and the percentage of cells in the Sub G0 phase is presented as the mean ± SD. * *p* < 0.05 (ANOVA followed by Tukey’s test).

Doxorubicin, EUD, and EUA extracts were used to treat the HeLa cells, and their results were compared to those of the cells that received only the vehicle (negative control). According to Figure 5 and Figure 6, it is possible to observe that the entire treatment can affect the cycle arrest of HeLa cells using flow cytometry with PI staining. In the G1/G0 phase, the negative control cells had 56.75%, while the cells treated with the positive control doxorubicin had 42.75%. On the other hand, 36% of the cells were treated with EUD extract, and 68.33% were treated with EUA in this phase. All the groups, when compared to the negative control, showed statistically significant differences (*p* < 0.05) (Figure 5).

In the G2/M phase, 7.97% of the negative control cells were in this stage of the cell cycle, and 0.245% of the doxorubicin-treated cells, 0.74% of the EUD-treated cells, and 2.36% of the EUA-treated cells were in this phase. All the groups showed statistically significant differences compared to the negative control group (Figure 5).

In the S phase, 30.55% of the negative control cells were in this stage of the cell cycle, while 12.1% of the cells treated with doxorubicin, 15.16% of the cells treated with EUD, and 24.81% of the cells treated with EUA were also in this phase; only the groups that received treatment with doxorubicin and EUD showed a statistically significant difference compared to the negative control (Figure 5).

In the SubG0 phase, the negative control cell percentage was 2.97%, the doxorubicin group was 44.20%, the EUD group was 46.52%, and the EUA group was 2.98%. The doxorubicin and EUD groups differed significantly, demonstrating the potential of these two treatments to induce cell death, as analyzed by the increase in DNA fragments (Sub-Go) (Figure 6).

## 3. Discussion

*Eugenia uniflora*, popularly known as pitanga, has been the focus of numerous studies due to its potential as a source of bioactive compounds, particularly molecules with anticancer activity. These studies are essential in identifying new therapeutic agents that can complement or potentially replace conventional treatments, which are often associated with significant adverse effects and increasing drug resistance [29].

*Eugenia uniflora* essential oils, particularly those rich in oxygenated sesquiterpenes and sesquiterpene hydrocarbons such as curzerene, have demonstrated cytotoxic activity against several cancer cell lines, including colon, gastric, and melanoma cancers. These oils show promise as candidates for developing anticancer herbal medicines [30]. In addition, the phenolic compounds present in *Eugenia uniflora* leaves, such as flavonoids and ellagitannins, have also shown cytotoxic activity against gastric adenocarcinoma (AGS) cells and anti-*Helicobacter pylori* activity [31]. The antiproliferative activity of these compounds appears to be associated with the bioactive properties of tannins and flavonoids. New sesquiterpenoids known as eugenilones have recently been identified in *E. uniflora*’s fruits. While these compounds have demonstrated significant anti-inflammatory effects, their potential anticancer activity remains to be thoroughly investigated [23].

Thus, this present study demonstrates the antiproliferative activity of *Eugenia uniflora* L. leaf extracts (EUH, EUD, and EUA) against HeLa cervical cancer cells, with the IC_50_ values indicating moderate to potent cytotoxicity. Among the three extracts, the dichloromethane fraction (EUD) exhibited the most powerful effect (IC_50_ = 33.79 μg/mL), followed by EUA (38.38 μg/mL) and EUH (63.03 μg/mL). These findings align with previous studies highlighting the cytotoxic potential of Myrtaceae species, which are known to contain bioactive secondary metabolites such as flavonoids, terpenes, and tannins [32]. In 2012, Ismiyati and collaborators demonstrated the antiproliferative potential of *E. uniflora* by testing the ethanolic extract on breast cancer cells, and this activity was associated with the presence of phenolic, flavonoid, and saponin compounds, which were suggested to be responsible for the antiproliferative effect [33].

The potent activity observed in the semi-polar (EUD) and polar (EUA) fractions suggests that compounds with intermediate polarity, such as flavonoid aglycones, sesquiterpenes, or triterpenoids, may be responsible for the observed bioactivity. This is supported by the spectrophotometric quantification of total phenolics and flavonoids, which showed a higher abundance of these classes in the more polar extracts, consistent with their solubility profiles. When we analyzed the antiproliferative activity of EUA and EUD, we observed that both extracts can inhibit cell proliferation at comparable concentrations. However, EUA demonstrated greater selectivity than EUD and exhibited a stronger ability to inhibit cell migration at the tested doses. These differences in cellular behavior were also evident in the cell cycle analysis. Treatment with EUA led to cell cycle arrest in the G0/G1 phase, indicating that many cells did not enter the division cycle. Furthermore, a reduction in the number of cells in the S phase was observed, suggesting that DNA replication was impaired in the EUA-treated cells.

In contrast, the cells treated with EUD exhibited slightly lower selectivity and a reduced ability to inhibit cell migration compared to EUA. Interestingly, EUD affected the cell cycle differently: a decrease in the G0/G1 phase was observed, like the action of the chemotherapeutic agent doxorubicin, along with reductions in the S and G2/M phases, and an increase in the SubG0 population, indicating DNA fragmentation and suggesting an increase in cell death.

Based on these analyses, it is evident that EUA and EUD exert distinct effects on tumor cells, inhibiting proliferation through different mechanisms. These differences may be attributed to the particular chemical compositions of the two extracts. Compounds such as galloyl hexoside, galloylquinic acid, gallic acid, myricetin-O-rhamnoside, and ellagic acid were identified in EUA but not in EUD, while sesquiterpenoids were present in both.

Several compounds identified in EUA have been reported to have anticancer activity. For example, ellagic acid has been shown to inhibit cell proliferation, induce cell cycle arrest at the G2 phase, and trigger apoptosis in SiHa cells, a cervical squamous cell carcinoma line [34]. It also inhibited the proliferation of non-small-cell lung cancer cells (NCI-H358 and A549) and breast cancer cells (MDA-MB-468 and MDA-MB-231). It interfered with SHP2-mediated signaling pathways, affecting critical cellular processes such as proliferation, migration, and apoptosis [35]. In 2023, two flavonol glycosides, myricitrin and its newly described isomer, named unifloratrin, were isolated and characterized from the ethyl acetate fraction of *E. uniflora* leaves. The compounds showed considerable cytotoxicity against human cervical (HeLa) and liver (Hep-G2) cancer cells, in vitro, with myricitrin (1) demonstrating better activity than unifloratrin (2) in HeLa cells, once again indicating that flavonoids may be responsible for the biological activity of the species [36].

In silico analyses have also suggested that quinic acid may possess anticancer potential in cervical cancer, acting on multiple targets within tumor cells [37]. Nevertheless, research on this topic remains limited. The sesquiterpenoids identified in this study have only recently been described in the literature, and, to date, their anti-inflammatory properties have been the only ones investigated. This class of compounds holds significant potential for exploration. Sesquiterpenoids, composed of 15 carbon atoms derived from three isoprene units, are commonly found in higher plants and exhibit diverse structures, including acyclic, mono-, bi-, tri-, and tetracyclic forms. These compounds are believed to play protective roles in plants against insect and microbial attacks and have demonstrated therapeutic potential in inhibiting cancer progression [38].

Therefore, the cytotoxic activity observed in HeLa cells may be attributed to the presence of a diverse set of bioactive compounds identified in the extracts of Eugenia uniflora, particularly phenolic acids, flavonoids, and rearranged sesquiterpenoids. Compounds such as gallic acid and ellagic acid have been extensively reported to induce apoptosis in cervical cancer cells through mechanisms involving p53/Bax activation, caspase cascade induction, and inhibition of pro-survival pathways like Akt and NF-κB. Similarly, quinic acid and its derivatives have shown pro-apoptotic and anti-angiogenic properties in other tumor models by modulating MAPK signaling and reducing MMP-9 expression [39]. Additionally, the sesquiterpenoids eugenilones and eugenunilones—recently characterized in *E. uniflora*—exhibit structural motifs commonly associated with cytotoxicity, such as α,β-unsaturated carbonyls and epoxide groups, suggesting a potential role in oxidative stress induction and apoptosis. Altogether, the combination of these constituents likely acts synergistically to reduce cell viability, promoting apoptotic pathways and cell cycle arrest. These findings support the hypothesis that *E. uniflora* extracts possess multiple classes of phytochemicals with complementary anticancer mechanisms, warranting further studies focused on compound isolation and mechanistic validation.

While this study provides promising evidence of the antiproliferative effects of *Eugenia uniflora* L. leaf extracts on cervical cancer cells, certain limitations should be acknowledged. First, the mechanisms underlying the observed cytotoxicity and cell cycle arrest were not fully elucidated at the molecular level. Although flow cytometry and migration assays suggest specific cellular responses, additional studies involving gene and protein expression analyses (e.g., apoptosis markers, cyclins, and migration-related proteins) are necessary to confirm the pathways involved. Second, the cytotoxicity of the extracts was evaluated in vitro; thus, in vivo studies are required to assess selectivity and therapeutic relevance. Finally, although LC-MS/MS enabled tentative identification of metabolites, the lack of compound isolation and structural confirmation by NMR limits the ability to correlate bioactivity with specific constituents precisely. Future research should focus on isolating active compounds, elucidating their mechanisms of action, and evaluating their efficacy and safety in preclinical models.

## 4. Materials and Methods

### 4.1. Material Plant and Preparation of Extracts

Leaves of *Eugenia uniflora* were collected in the mountainous region of Nova Friburgo, in the state of Rio de Janeiro (RJ), Brazil, geographic coordinates −22,276,061, −42,535,962, in April 2022. The plant material was dried in a circulating air oven at 55 °C for 72 h. Subsequently, the dried leaves were pulverized and subjected to successive maceration using one organic solvent at a time, in order of increasing polarity: hexane, dichloromethane, and ethyl acetate (Dinâmica^®^, São Paulo, Brazil. Each solvent was used separately to obtain the corresponding extracts, which were then concentrated under reduced pressure using a rotary evaporator, yielding the following fractions: EUH (*E. uniflora* hexane extract), EUD (*E. uniflora* dichloromethane extract), and EUA (*E. uniflora* ethyl acetate extract).

### 4.2. Determination of Total Soluble Flavonoid Content

The EUD and EUA extracts were weighed and dissolved in ethanol to a final concentration of 2 mg/mL. Quercetin (Sigma-Aldrich^®^, St Louis, MO, USA) was used as the reference standard and prepared from a stock solution of 2 mg/mL, followed by serial dilution to obtain concentrations ranging from 6 µg/mL to 200 µg/mL to construct the calibration curve. In a 96-well microplate, the following were added: 100 µL of Milli-Q water, 60 µL of ethanol, 10 µL of aluminum chloride solution (40 mg/mL in water), 10 µL of sodium acetate solution (54.46 mg/mL in water), 20 µL of each quercetin standard (from the most diluted to the most concentrated), and 20 µL of each sample extract. The plate was incubated at room temperature for 30 min, and the absorbance was subsequently measured at 415 nm using a microplate spectrophotometer. The results were expressed as micrograms of quercetin equivalent per milligram of extract (µg QE/mg). All the analyses were performed in triplicate [20].

### 4.3. Determination of Total Soluble Phenolic Content

The EUD and EUA extracts were also solubilized in ethanol at a concentration of 2 mg/mL. An analytical curve was prepared using gallic acid (Sigma-Aldrich^®^) as the standard, with serial dilutions ranging from 6 µg/mL to 200 µg/mL. In a 96-well microplate, 26 µL of Folin–Ciocalteu reagent, 26 µL of sodium carbonate (Na_2_CO_3_), and 182 µL of Milli-Q water were added to the samples, followed by incubation for 2 h in the absence of light. Absorbance was then measured at 726 nm using a spectrophotometer. The results were expressed as micrograms of gallic acid equivalent per milligram of extract (µg GAE/mg). All experiments were conducted in triplicate [20].

### 4.4. Chemical Profile Analysis of the Samples by LC-MS/MS

Samples of EUD and EUA (2 mg) were dissolved in 1 mL of a solution containing methanol and formic acid (H_3_OH:HCOOH, 0.1%), centrifuged for 10 min, and subsequently injected into the liquid chromatography system. The analysis was conducted using an ultra-high-performance liquid chromatography (UHPLC) system (Shimadzu^®^, Kyoto, Japan) coupled to a microOTOF-Q II mass spectrometer (Bruker Daltonics, Billerica, MA, USA) equipped with an electrospray ionization (ESI) source for ESI-HRMS/MS analysis.

Chromatographic separation was performed using a Shimadzu XR-ODS C18 analytical column (75 mm × 2.1 mm, 2.1 µm; Shimadzu, Kyoto, Japan). Sample injections (20 µL) were carried out using an autosampler. The mobile phase consisted of 0.1% formic acid in water (solvent A) and methanol (solvent B). The elution gradient was as follows: 0.0–16.0 min (40–70% B); 16.0–40.0 min (70–100% B); 46.0–50.0 min (100% B); 50.0–55.0 min (100–40% B); and 55.0–60.0 min (40% B). The flow rate was maintained at 0.4 mL/min. The temperature of the column is 20 °C.

Instrumental parameters were set as follows: capillary voltage of 4.5 kV, ESI operated in both positive and negative ionization modes, end plate offset at 500 V, nebulizer pressure at 40 psi, dry gas (N_2_) flow at 8 mL/min, and a temperature of 200 °C. Collision-induced dissociation (CID) was performed in auto MS/MS mode. Mass spectra were acquired over the *m*/*z* range of 100–1200.

### 4.5. Cell Viability Assay

The HeLa (cervical cancer) and NIH/3T3 (murine fibroblast) cells were seeded in 96-well plates at a density of 3 × 10^4^ cells/well in DMEM (Dulbecco’s Modified Eagle Medium; Gibco, Thermo Fisher, Paisley, UK), supplemented with 10% fetal bovine serum (FBS; Cultilab^®^, São Paulo, Brazil) and 1% penicillin/streptomycin (Pen/Strep—Cultilab^®^, Campinas, Brazil). The cells were incubated at 37 °C in a humidified atmosphere containing 5% CO_2_ for 24 h. After incubation, the cells were treated with EUD and EUA extracts at concentrations ranging from 3.5 µg/mL to 250 µg/mL and incubated for an additional 48 h. The cell lines were kindly donated by Cemib (Multidisciplinary Center for Biological Research in the Laboratory Animal Science Area, Campinas, Brazil)—Unicamp/SP.

One row of wells was treated only with the vehicle (culture medium) to serve as the negative control, representing 100% cell viability. Doxorubicin (Libbs^®^, São Paulo, Brazil) and carboplatin (Blau Farmacêutica^®^, São Paulo, Brazil) were used as the positive controls.

Following treatment, the medium was removed, and 100 µL/well of MTT reagent (5 mg/mL; 3-[4,5-dimethylthiazol-2-yl]-2,5-diphenyltetrazolium bromide; Invitrogen, Thermo Fisher, Waltham, MA, USA) was added. The plates were incubated for 4 h at 37 °C. After incubation, the resulting formazan crystals formed in viable cells were solubilized by adding 100 µL/well of DMSO (dimethyl sulfoxide; Labsynth, São Paulo, Brazil). Absorbance was measured at 570 nm using an Epoch-Biotek ELISA microplate spectrophotometer (BioTek Instruments, Winooski, VT, USA).

Statistical analysis was performed using GraphPad Prism 8.4.6 (GraphPad Software, Inc., Boston, MA, USA) via one-way ANOVA followed by Tukey’s post hoc test. Differences were considered statistically significant at *p* < 0.05. All the experiments were conducted in triplicate [3].

### 4.6. Selectivity Index (SI) Calculation

The selectivity index (SI) was calculated by dividing the IC_50_ value of NIH/3T3 cells by the IC_50_ of HeLa cells. Samples with SI ≥ 2 were considered selectively cytotoxic to cancer cells [3].

### 4.7. Cell Migration Assay

The HeLa cells (70,000–80,000 cells/well) were seeded in 24-well plates with DMEM supplemented with 10% FBS and 1% Pen/Strep and incubated at 37 °C with 5% CO_2_ until 80% confluence. The medium was replaced with DMEM containing 0.2% FBS, and after 24 h, a scratch was made in the cell monolayer using a 1000 µL pipette tip. The cells were washed with sterile PBS, and they were treated with doxorubicin (positive control) and EUD and EUA extracts at ¼ of their IC_50_ values: 7.5 µg/mL (EUD), 10 µg/mL (EUA), and 0.375 µg/mL (doxorubicin).

Images were captured at 0 h (T0), 12 h (T1), 18 h (T2), and 24 h (T3) using an EVOS XL Core Imaging System microscope (10× objective). Scratch area percentages were analyzed using the ImageJ software 1.54g/Java 1.8.0_421 (32-bit), and statistical analysis was performed using one-way ANOVA with GraphPad Prism 8.4.3. All the experiments were conducted in triplicate [3].

### 4.8. Cell Cycle Analysis by Flow Cytometry

The HeLa cells (2 × 10^5^ cells/well) were seeded in 6-well plates and incubated for 24 h. The cells were then treated with the EUD and EUA extracts and doxorubicin at their respective IC_50_ concentrations. The vehicle-treated cells served as the negative control. After 48 h, cells were harvested with trypsin, centrifuged, fixed in 70% ethanol, and stained with propidium iodide (PI) (Sigma-Aldrich^®^). The samples were incubated at 37 °C for 30 min in the dark and analyzed using a BD Accuri™ C6 Plus flow cytometer. Histograms were processed with the Cell Quest Pro software (version 5.1). Data was analyzed using two-way ANOVA in GraphPad Prism 8.4.3, with significance at *p* < 0.05.

## 5. Conclusions

The present study demonstrated that the EUD and EUA extracts of *Eugenia uniflora* exhibit significant cytotoxic effects against cervical cancer cells, inducing cell cycle arrest and inhibiting cell migration. These biological activities are likely associated with the presence of flavonoids and phenolic compounds identified in the extracts. Among the tested fractions, EUD showed the highest cytotoxicity and flavonoid content. At the same time, EUA presented the highest total phenolic content, suggesting that both classes of metabolites contribute to the observed effects.

These findings highlight the therapeutic potential of *E. uniflora* as a source of bioactive compounds for complementary cancer treatments. Future studies should aim to isolate and characterize the individual active constituents, elucidate their molecular mechanisms of action, and assess their efficacy in vivo models. Such investigations may support the development of novel phytotherapeutic strategies for cervical cancer, a disease that remains a significant cause of morbidity and mortality among women worldwide.

## Figures and Tables

**Table 1:** Total soluble phenolic and total flavonoid content of the fractions *Eugenia uniflora* dichloromethane (EUD) and *Eugenia uniflora* ethyl acetate (EUA).

Samples	Total Soluble Phenolic (μg GAE/mg)	Total Flavonoid (μg E Quercetin/mg)
EUD	10.71 ± 4.29	23.82 ± 1.31
EUA	45.94 ± 8.25	14.22 ± 3.44

The results are expressed as the mean of the triplicate followed by the standard deviation; μg GAE/mg: total soluble phenolics expressed in terms of micrograms of gallic acid equivalents per milligram of extract or fraction on a dry basis; μg quercetin/mg: total flavonoids expressed in terms of microgram quercetin equivalents per milligram of extract or fraction on a dry basis.

**Table 2:** Annotation of compounds tentatively identified by HPLC-HRESIMS in *Eugenia uniflora*.

Peak No.	t_R_ (min)	*m*/*z* [M + H]^+^	*m*/*z* [M + Na]^+^	*m*/*z* [M − H]^−^	Molecular Formula	Error (ppm)	MS/MS	Tentative Assignment	Ext	Reference
1	1.7	-	-	191.0560	C_7_H_12_O_6_	−5.0	127	Quinic acid	A	[20]
	1.7	-	-	331.0671	C_13_H_16_O_10_	−3.4	221; 169; 125	Galloyl hexoside	A	[20,21]
2	1.7	-	-	361.0768	C_14_H_18_O_11_	−0.7	191; 169; 125	Galloylquinic acid	A	[20]
3	2.3	-	-	169.0139	C_7_H_6_O_5_	−4.4	125	Gallic acid	A	[20,21]
4	8.3	-	-	463.0877	C_21_H_20_O_12_	−1.3	317; 316; 271	Myricetin-O-rhamnoside	A	[20]
5	10.3	-	-	300.9981	C_14_H_6_O_8_	−0.7	284; 257; 229; 201; 173	Ellagic acid	A	[21]
6	10.8	247.1318	-	-	C_15_H_18_O_3_	4.3	145; 119; 105; 93	Eugenunilone D or Eugenilone F	D and A	[22,23]
7	10.8	265.1424	-	-	C_15_H_20_O_4_	3.9	187; 164; 131; 125; 107; 91	Eugenilone L I	D and A	[22,23]
8	11.6	-	291.1580	-	C_17_H_22_O_4_	4.4	257; 147; 119; 105; 91	NI	D and A	[22,23]
9	11.6	-	305.1371	-	C_17_H_20_O_5_	4.1	185; 151; 114; 105;	NI	D and A	[22,23]
10	12.3	245.1178	-	-	C_15_H_16_O_3_	−2.4	201; 156; 129; 105; 91	NI	D and A	[22,23]
11	12.3	263.1283	-	-	C_15_H_18_O_4_	−2.0	193; 156; 147; 105; 91	Eugenilone B	D and A	[22,23]
12	13.0	247.1339	-	-	C_15_H_18_O_3_	−4.2	145; 131; 119; 105; 93	Eugenunilone D or Eugenilone F	D and A	[22,23]
13	13.0	265.1445	-	-	C_15_H_20_O_4_	−4.0	229; 187; 164; 131; 125; 107; 91	Eugenilone L II	D and A	[22,23]
14	14.1	249.1497	-	-	C_15_H_20_O_3_	−4.7	185; 143; 131; 119; 105; 91	Eugenunilone C or Eugenilone A or E	D and A	[22,23]
15	14.1	265.1428	-	-	C_15_H_20_O_4_	2.4	229; 179; 131; 119; 105; 91	Eugenilone L III	D and A	[22,23]
16	15.6	249.1499	-	-	C_15_H_20_O_3_	−5.5	185; 143; 131; 119; 105; 91	Eugenunilone C or Eugenilone A or E	D and A	[22,23]
17	15.6	231.1370	-	-	C_15_H_18_O_2_	4.1	155; 128; 119; 105; 91	Eugenunilone F or H	D and A	[22,23]
18	17.0	289.1420	-	-	C_17_H_20_O_4_	5.0	213; 179; 128; 119; 105; 91	NI	D and A	[22,23]
19	18.2	245.1174	-	-	C_15_H_16_O_4_	−0.7	171; 156; 119; 105; 91	NI	D and A	[22,23]
20	18.2	307.1550	-	-	C_17_H_22_O_5_	−3.3	183; 143; 117; 105; 91	Eugenilone M or N	D and A	[22,23]
21	18.7	249.1493	-	-	C_15_H_20_O_3_	−3.1	185; 143; 131; 128; 119; 105; 91	Eugenunilone C or Eugenilone A or E	D and A	[22,23]
22	20.4	247.1336	-	-	C_15_H_18_O_3_	−3.0	147; 131; 119; 105; 91	Eugenunilone D or Eugenilone F	D and A	[22,23]
23	20.4	307.1551	-	-	C_17_H_22_O_5_	−3.6	187; 159; 131; 105; 91	Eugenilone M or N	D and A	[22,23]
24	22.6	231.1392	-	-	C_15_H_18_O_2_	−5.4	209; 155; 142; 128; 119; 105; 91	Eugenunilone F or H	D and A	[22,23]
25	22.6	291.1599	-	-	C_17_H_22_O_4_	−2.8	213; 157; 142; 128; 119; 105; 91	NI	D and A	[22,23]
26	24.7	291.1603	-	-	C_17_H_22_O_4_	−4.2	213; 145; 105; 91	NI	D and A	[22,23]
27	26.4	289.1450	-	-	C_17_H_20_O_4_	−5.4	229; 183; 155; 109; 91	NI	D and A	[22,23]
28	26.4	329.1401	-	-	C_19_H_20_O_5_	−5.3	229; 183; 155; 149; 109;	NI	D and A	[22,23]

I, II, and III—numbers used to discriminate putative individual isomers. NI—not identified compounds. Ext = extract; A = ethyl acetate extract; D = dichloromethane extract.

**Table 3:** IC_50_ values and selectivity index (SI) of the EUD and US samples, as well as of the positive controls doxorubicin and carboplatin, in the Hela and non-tumor NIH/3T3 tumor cells.

Samples	IC_50_ Tumor Cells HeLa (μg/mL)	IC_50_ Non-Tumor cells NIH/3T3 (μg/mL)	Selectivity Index (SI)
EUH	63.03	>100	>6.3
EUD	33.75	>100	>2.96
EUA	38.38	>100	>2.60
Doxorubicin	1.591	2.321	1.5
Carboplatin	52.40	38.32	0.73

## Data Availability

TThe original contributions presented in this study are included in the article. Further inquiries can be directed to the corresponding author.
